# Synergistic effects of childhood adversity and polygenic risk in first-episode psychosis: the EU-GEI study

**DOI:** 10.1017/S0033291721003664

**Published:** 2023-04

**Authors:** Monica Aas, Luis Alameda, Marta Di Forti, Diego Quattrone, Paola Dazzan, Antonella Trotta, Laura Ferraro, Victoria Rodriguez, Evangelos Vassos, Pak Sham, Giada Tripoli, Caterina La Cascia, Daniele La Barbera, Ilaria Tarricone, Roberto Muratori, Domenico Berardi, Antonio Lasalvia, Sarah Tosato, Andrei Szöke, Pierre-Michel Llorca, Celso Arango, Andrea Tortelli, Lieuwe de Haan, Eva Velthorst, Julio Bobes, Miguel Bernardo, Julio Sanjuán, Jose Luis Santos, Manuel Arrojo, Cristina Marta Del-Ben, Paulo Rossi Menezes, Jean-Paul Selten, Peter B. Jones, Hannah E. Jongsma, James B. Kirkbride, Bart P. F. Rutten, Jim van Os, Charlotte Gayer-Anderson, Robin M. Murray, Craig Morgan

**Affiliations:** 1 Department of Psychosis Studies, Institute of Psychiatry, Psychology, and Neuroscience, King's College London, London SE5 8AF, UK; 2 Norment, Oslo University Hospital, Oslo, Norway; 3 Service of General Psychiatry, Treatment and Early Intervention in Psychosis Program, Lausanne University Hospital (CHUV), Lausanne, Switzerland; 4 Instituto de Investigación Biomédica de Sevilla, Universidad de Sevilla, Seville, Spain; 5 Department of Psychiatry, Hospital Universitario Virgen del Rocío, Universidad de Sevilla, Sevilla, Spain; 6 Social, Genetic and Developmental Psychiatry Centre, Institute of Psychiatry, Psychology and Neuroscience, King's College London, London SE5 8AE, UK; 7 Biomedicine, Neuroscience and Advanced Diagnostic (BiND) Department, University of Palermo, Palermo, Italy; 8 Department of Psychiatry, University of Hong Kong, Pok Fu Lam, Hong Kong; 9 Department of Medical and Surgical Science, Psychiatry Unit, Alma Mater Studiorum Università di Bologna, 40126 Bologna, Italy; 10 Department of Mental Health and Pathological Addiction, Bologna Local Health Authority, Bologna, Italy; 11 Department of Biomedical and Neuro-motor Sciences, Psychiatry Unit, Alma Mater Studiorum Università di Bologna, 40126 Bologna, Italy; 12 Section of Psychiatry, Department of Neuroscience, Biomedicine and Movement Sciences, University of Verona, Piazzale L.A. Scuro 10, 37134 Verona, Italy; 13 INSERM U955, Equipe 15, Institut National de la Santé et de la Recherche Médicale, 94010 Créteil, France; 14 CMPB CHU Clermont-Ferrand, EA 7280, University Clermont Auvergne, Clermont-Ferrand, France; 15 Department of Child and Adolescent Psychiatry, Institute of Psychiatry and Mental Health, Hospital General Universitario Gregorio Marañón, School of Medicine, Universidad Complutense, IiSGM (CIBERSAM), 28007 Madrid, Spain; 16 Etablissement Public de Santé Maison Blanche, 75020 Paris, France; 17 Department of Psychiatry, Early Psychosis Section, Amsterdam UMC, Location: Academic Medical Centre, University of Amsterdam, 1105 AZ Amsterdam, The Netherlands; 18 Department of Psychiatry, Icahn School of Medicine, Mount Sinai, NY, USA; 19 Faculty of Medicine and Health Sciences – Psychiatry, Universidad de Oviedo, ISPA, INEUROPA, CIBERSAM, 33006 Oviedo, Spain; 20 Barcelona Clinic Schizophrenia Unit, Hospital Clinic of Barcelona, University of Barcelona; IDIBAPS, CIBERSAM, 08036 Barcelona, Spain; 21 Department of Psychiatry, School of Medicine, Universidad de Valencia, Centro de Investigación Biomédica en Red de Salud Mental (CIBERSAM), 46010 Valencia, Spain; 22 Department of Psychiatry, Servicio de Psiquiatría Hospital ‘Virgen de la Luz’, 16002 Cuenca, Spain; 23 Department of Psychiatry, Psychiatry Genetic Group, Instituto de Investigación Sanitaria de Santiago de Compostela, Complejo Hospitalario Universitario de Santiago de Compostela, 15706 Santiago de Compostela, Spain; 24 Division of Psychiatry, Department of Neuroscience and Behaviour, Ribeirão Preto Medical School, University of São Paulo, São Paulo 14049-900, Brazil; 25 Department of Preventative Medicine, Faculdade de Medicina FMUSP, University of São Paulo, São Paulo 01246-903, Brazil; 26 Rivierduinen Institute for Mental Health Care, 2333 ZZ Leiden, The Netherlands; 27 Department of Psychiatry, University of Cambridge, Cambridge CB2 0SZ, UK; 28 Psylife Group, Division of Psychiatry, University College London, London W1T 7NF, UK; 29 Department of Psychiatry and Neuropsychology, School of Mental Health and Neuroscience, South Limburg Mental Health Research and Teaching Network, Maastricht University Medical Centre, 6200 MD Maastricht, The Netherlands; 30 Health Service and Population Research, Institute of Psychiatry, Psychology, and Neuroscience, King's College London, London SE5 8AF, UK

**Keywords:** Childhood trauma, first-episode psychosis, interaction contrast ratio, polygenic risk, schizophrenia, synergistic effects

## Abstract

**Background:**

A history of childhood adversity is associated with psychotic disorder, with an increase in risk according to the number of exposures. However, it is not known why only some exposed individuals go on to develop psychosis. One possibility is pre-existing polygenic vulnerability. Here, we investigated, in the largest sample of first-episode psychosis (FEP) cases to date, whether childhood adversity and high polygenic risk scores for schizophrenia (SZ-PRS) combine synergistically to increase the risk of psychosis, over and above the effect of each alone.

**Methods:**

We assigned a schizophrenia-polygenic risk score (SZ-PRS), calculated from the Psychiatric Genomics Consortium (PGC2), to all participants in a sample of 384 FEP patients and 690 controls from the case–control component of the EU-GEI study. Only participants of European ancestry were included in the study. A history of childhood adversity was collected using the Childhood Trauma Questionnaire (CTQ). Synergistic effects were estimated using the interaction contrast ratio (ICR) [odds ratio (OR)_exposure and PRS_ − OR_exposure_ − OR_PRS_ + 1] with adjustment for potential confounders.

**Results:**

There was some evidence that the combined effect of childhood adversities and polygenic risk was greater than the sum of each alone, as indicated by an ICR greater than zero [i.e. ICR 1.28, 95% confidence interval (CI) −1.29 to 3.85]. Examining subtypes of childhood adversities, the strongest synergetic effect was observed for physical abuse (ICR 6.25, 95% CI −6.25 to 20.88).

**Conclusions:**

Our findings suggest possible synergistic effects of genetic liability and childhood adversity experiences in the onset of FEP, but larger samples are needed to increase precision of estimates.

## Introduction

Psychotic disorders such as schizophrenia (SZ) have detrimental societal, economic and individual costs (Charlson et al., 2018). A history of childhood adversity is one of the strongest environmental predictors of mental illness, crossing boundaries of affective and psychotic illnesses (van, van, Myin-Germeys, & van, 2013). A history of adversity is associated with up to a three-fold life-long increased risk of psychotic disorder with an increase in risk according to the number and severity of exposures (Aas et al., 2016; Croft et al., 2019; Varese et al., 2012). However, it is not known why only some exposed individuals go on to develop psychosis. One plausible explanation is that exposed individuals differ in their pre-existing biological vulnerability to psychosis, characterised by several genetic variants with small effect sizes (Schizophrenia Working Group of the Psychiatric Genomics Consortium, , 2014; Tesli et al., 2014; Zheutlin et al., 2019). However, it is yet to be determined if both high polygenic risk and childhood trauma increase the risk above that of each alone (additive synergistic effects).

Initial studies investigating interactions between exposure to childhood adversities and underlying genetic susceptibility in schizophrenia focused mainly on candidate genes, including *AKT1*, *COMT*, *BDNF*; findings have been inconclusive (Aas et al., 2014; Modinos et al., 2013; Trotta et al., 2019). Studying single candidate genes may miss important aspects of the aetiology of psychoses, as psychotic disorders are polygenic in nature (Arango, 2017; Vassos et al., 2017).

As a consequence of this limitation, studies using polygenic risk score (PRS) have emerged. Schizophrenia PRSs are calculated by using subsets of single-nucleotide polymorphisms (SNPs) from large schizophrenia cases and healthy control genome-wide association studies. PRS are selected according to their *p* value and weighted by their effect size to calculate a PRS for each individual in an independent validation sample. The PRS can then be tested for its ability to differentiate between cases and controls in the validation dataset (Dudbridge, 2013; Pardiñas et al., 2019; Purcell et al., 2009). The PRS explains around 7% of the variation in the liability for schizophrenia assuming a lifetime risk of 1% (Pardiñas et al., 2019; Schizophrenia Working Group of the Psychiatric Genomics Consortium, 2014). The development of PRS techniques has opened a new avenue for studying overall genetic susceptibility. Such a PRS can, in turn, be used to study interaction effects with environmental risk factors.

In major depressive disorder (MDD), findings of interactions between polygenic risk for MDD and childhood adverse events have been mixed (Mullins et al., 2016; Peyrot et al., 2014, 2017), with the most recent and largest study not providing evidence for interaction effects (Peyrot et al., 2017). A recent study of those with a long-standing diagnosis of schizophrenia provided some evidence of synergistic effects; that is the combined effect of polygenic risk and childhood trauma was greater than the sum of their individual effects (Guloksuz et al., 2019). In first-episode psychosis (FEP), to our knowledge, only one pilot study (*N* < 200) to date has investigated interaction between childhood trauma and polygenic risk scores for schizophrenia (SZ-PRS) (Trotta et al., 2016), concluding that higher SZ-PRS and childhood adversities each predicted case status independent of each other with no strong evidence of interactions. However, the sample was relatively small and synergistic effects using the interaction contrast ratio (ICR) or the relative excess risk due to an interaction was not reported, hence further studies are clearly needed.

Therefore, the current study aimed to investigate synergistic (combined) effects of SZ-PRSs and childhood adversities in the FEP status in a large (*N* < 1000) multi-centre study (EU-GEI). Although synergistic effects of trauma and polygenic risk have been published in a chronic sample of schizophrenia within the EU-GEI (20), this is the first time this is investigated in FEP. We investigated synergistic effects by estimating the ICR for polygenic risk and childhood adversity on the risk of developing an FEP diagnosis. The ICR provides a measure of interaction on the additive scale by quantifying the combining effect two exposures over and above the effect of each alone (Hilker et al., 2018). It has been argued that additive interactions most closely correspond to mechanistic interactions and are specifically useful to test biological interactions (VanderWeele & Knol, 2014).

Our hypothesis was that the combined effect on odds of psychosis of the two exposures (polygenic risk and a history of childhood adverse events) would be greater than the sum of their individual effects.

## Methods and materials

### Study design and participants

The sample was drawn from the EU-GEI (European Network of National Schizophrenia Networks Studying Gene–Environment Interactions) multi-centre study. The EU-GEI study is a multi-centre incidence and case–sibling–control study of genetic and environmental determinants of psychotic disorders (Di Forti et al., 2019; Gayer-Anderson et al., 2020; Jongsma et al., 2018; Quattrone et al., 2019). The current study was based on participants from work package 2 of the EU-GEI study ‘Incidence and first-episode’ (see Di Forti et al. 2019; Mallett, Leff, Bhugra, Pang, & Zhao, 2002 for more details). For the analyses presented in this paper, only participants of European ancestry (see below) and who had complete data on SZ-PRS and childhood adverse events were included. Patients and controls were recruited from 16 different sites as part of the EU-GEI study (for an overview of recruitment, see online Supplementary Tables S1 and S2). Cases and controls were not related.

Participants, aged 18–64 years, were invited to take part in the study if they presented to mental healthcare services during the case ascertainment period for a first-episode of psychosis. The diagnosis was confirmed by the Operational Criteria Checklist for Psychotic and Affective Illness within in the EU-GEI consortia (McGuffin, Farmer, & Harvey, 1991; Quattrone et al., 2019). As described by Gayer-Anderson et al. (2020) research teams were overseen by a psychiatrist with experience in epidemiological research and included trained research nurses and clinical psychologists. Teams received training in epidemiological principles and incidence study design to minimise non-differential ascertainment bias across different local and national health care systems.

Patients were identified by clinically trained researchers who carried out regular checks across the 16 catchment areas. Exclusion criteria included previous treatment for psychosis, and a diagnosis of organic psychosis (ICD-10: F09) or transient psychotic symptoms resulting from acute intoxication (ICD- 10: F1X.5), and language barriers.

Control participants without a lifetime psychotic disorder were recruited from the same population as the cases using guided random and quota sampling strategies. Exclusion criteria for both controls and cases included intelligence quotient <70. Written informed consent was obtained from those who agreed to participate in the case–control study and an institutional review board (IRB) approval was obtained from all centres.

### Sociodemographic

Information on demographics, premorbid characteristics and social circumstances were collected from cases and controls using the Medical Research Council (MRC) Socio-demographic Schedule modified version (Mallett et al., 2002).

### Childhood Trauma Questionnaire (CTQ)

To measure adverse childhood events, we used the Childhood Trauma Questionnaire (CTQ) (Bernstein et al., 1994). The CTQ is a retrospective questionnaire enquiring about potentially traumatic experiences in childhood with answers ranging from ‘never true’, through ‘rarely true’, ‘sometimes true’, ‘often true’, to ‘very true’, yielding a total score, as well as five sub-scores: physical abuse, emotional abuse, sexual abuse, physical neglect and emotional neglect. The reliability and validity of the CTQ have been demonstrated previously. Data were dichotomised for each childhood adversity domain (0 = ‘absent’ and 1 = ‘present’), based on the moderate to severe cut-off score from the CTQ Manual (Bernstein et al., 1994) using the following cut-off scores for each domain: ≥13 for emotional abuse; ≥10 for physical abuse; ≥8 for sexual abuse; ≥15 for emotional neglect and ≥10 for physical neglect. Sensitivity analyses were conducted using the CTQ as a continuous measure following the procedures from the CTQ Manual with scores ranging from 25 to 125 (Bernstein et al., 1994).

### Genotyping and polygenic risk calculations

Samples were genotyped at the MRC Centre for Neuropsychiatric Genetics and Genomics in Cardiff (UK) using a custom Illumina HumanCoreExome-24 BeadChip genotyping array covering 570 038 genetic variants. To identify ethnic groups, we combined our dataset with the 1000 Genome Project (1000G), phase 3 and performed principal components analysis (PCA) on the overlapping SNPs. Only people of European ancestry were included in this study. Individuals of European ancestry were defined as having principal component (PC) values within 6 standard deviations from the mean PC of the EUR in 1000G, and retained for the downstream analyses, consistent with standard practice. SZ-PRS were generated using PRSice from the summary results of the PGC analysis of schizophrenia, wave 2 (Schizophrenia Working Group of the Psychiatric Genomics Consortium, 2014). Clumping was performed to obtain SNPs in approximate linkage disequilibrium with an *r*^2^ < 0.25 within a 250 kb window. PRSs were calculated within Europeans only and at *p* value thresholds of 0.05 (Schizophrenia Working Group of the Psychiatric Genomics Consortium, 2014). We used a *p*-threshold of 0.05 as this has shown to maximally capture polygenic risk across a large number of independent samples (Schizophrenia Working Group of the Psychiatric Genomics Consortium, 2014). Furthermore, each PRS was standardised to a mean of zero and standard deviation of 1, excluding the MHC region (Lewis & Vassos, 2017). SNPs within the extended MHC locus were excluded due to high levels of linkage disequilibrium in the region, as were insertion/deletion polymorphisms and ambiguous flip SNPs. In addition to only included Europeans we additionally corrected for genetic variation within the sample (population stratification) adjusting for 10 PCA.

### Statistics

The main analyses were carried out using Statistical Package for Social Sciences, Version 25.0 (SPSS Inc.). Logistic regression was used to estimate the odds of psychotic disorder (i.e. case status) by childhood adverse events and SZ-PRS. Moderate to severe childhood adversity was categorised as having at least one type of trauma reaching moderate to the severe level (Bernstein et al., 1994). The cumulative effect of childhood adversity (zero, one or two or more types of trauma) was categorised using moderate to severe cut-off score from the CTQ Manual (Bernstein et al., 1994) as previously described. Sensitivity analyses were conducted by analysing childhood adversity as a continuous variable dividing into subtypes of trauma.

The association between the SZ-PRS and the presence or absence of (i) psychotic disorder and (ii) childhood adversity (i.e. gene–environment correlation) was tested using a linear regression model, controlling for population stratification (adjusting for 10 PCA), sex, age and education level, because such factors could potentially bias the results (Trotta et al., 2016). Cases and controls were analysed separately.

We assessed for synergistic effects whether the combined effects of SZ-PRS and childhood adversity were greater than the sum of each effect alone (i.e. interaction on the additive scale) using the ICRs (Knol & VanderWeele, 2012; Knol, van der Tweel, Grobbee, Numans, & Geerlings, 2007). Using odds ratios (ORs) derived from logistic models, the ICR is estimated as OR_exposure and PRS_ − OR_exposure_ − OR_PRS_ + 1. An ICR greater than zero indicates a positive deviation from additivity (Knol & VanderWeele, 2012). For these analyses, SZ-PRS was dichotomised into two groups (below or 75th percentile and above) using the same method as described in Guloksuz et al. (2019), and data on childhood adversity were analysed using the predefined < or ⩾ moderate to severe cut-off scores described in the CTQ Manual (Bernstein et al., 1994) (see previous section). The confidence intervals (CIs) for the ICRs for each model were calculated using the delta method (Hosmer, & Lemeshow, 1992). To test the joint effects of environmental exposures and genetic score, we entered the four states occasioned by the combination of each exposure and binary SZ-PRS risk state as independent variables (three dummy variables), and case–control status as the dependent variable, in multilevel logistic regression models. Analyses were adjusted for site, sex, age and 10 PCs (covariates added into the logistic regression model). Sensitivity analyses were conducted examining PRS × childhood adversity additive interaction models analysing PRS as a continuous variable using a residual score of the PRS regressing out the effect of site, age, sex and 10 PCs following the principles described by VanderWeele and Knol (2014). The cumulative effect of childhood adversity (zero, one or two or more types of trauma) was categorised using moderate to severe cut-off score from the CTQ Manual (see description above), as well as dividing into trauma subtypes.

## Results

Sample characteristics are shown in Table 1. The sample was comprised of 384 FEP cases and 690 controls. Compared with controls, cases had a lower level of education. Patients were also more likely to be men and younger than the control group (see Table 1).

**Table 1.** Sample characteristics

	Cases *N* = 384	Control *N* = 690	Statistics
Sex			χ^2^ = 20.8, df = 1, *p* < 0.001
Men, *N* (%)	236 (61.5)	324 (46.9)	
Women, *N* (%)	148 (38.5)	366 (53.1)	
Age, mean ± s.d.	31.8 ± 10.9	38.2 ± 13.4	*F* = 62.4, df = 1, *p* < 0.001
Years of education, mean ± s.d.	13.4 ± 3.9	15.2 ± 4.1	*F* = 48.9, df = 1, *p* < 0.001

Both patients and controls were recruited from 16 different sites. Eight participants had missing data on years of education, and one participant had missing data on age. All participants had data on sex. All participants were Caucasians.

### Childhood adversity and case–control status

Cases reported more childhood adversities compared to controls (see Table 2). In total, 44% of the cases reported at least one moderate to severe form of childhood adversity compared to 21% of the controls. Cases were four times more likely to report two or more childhood adversities than controls and the OR was higher for multiple (OR 4.70; 95% CI 3.06–7.24; *p* < 0.001) than for single adverse childhood experiences (OR 2.41; 95% CI 1.44–4.00; *p* = 0.001, see Table 2). Emotional neglect was the most prevalent form of adversity in both cases [*N* = 93 (24%)] and in controls [*N* = 77 (11%)]. Sensitivity analysis of childhood adverse events as a continuous variable confirmed the association with increasing amounts adversity and case–control status (see online Supplementary Table S3), with similar OR across subtypes of trauma (see online Supplementary Table S4).

**Table 2.** Prevalence of childhood adversities amongst FEP cases and unaffected controls

Total adversity exposure	Cases *N* = 384	Controls *N* = 690	Unadjusted OR	95% CI	*p*	Adjusted OR^a^	95% CI	*p*
None, *N* (%)	213 (55.5)	543 (78.7)	1	–	–	1	–	–
One type, *N* (%)	79 (20.6)	96 (13.9)	2.19	1.39–3.45	<0.001	2.41	1.44–4.00	0.001
Two or more types, *N* (%)	92 (24.0)	51 (7.4)	4.60	3.17–6.71	<0.001	4.70	3.06–7.24	<0.001

CI, confidence interval; OR, odds ratio.

Childhood adversity was measured by the CTQ dichotomised into at least one type of trauma reaching moderate to severe levels, or two or more of traumas reaching moderate to severe levels based on predefined cut-off scores from the CTQ manual (Bernstein et al., 1994).

a Adjusted for site, sex, age at interview and years of education.

### Polygenic risk and case–control status

A higher PRS was associated with the FEP case status (OR 1.79; 95% CI 1.53–2.10; *p* < 0.001), which held when restricted to cases diagnosed with ICD-10 Schizophrenia Spectrum disorders (OR 1.98; 95% CI 1.65–2.38; *p* < 0.001). The relationship between PRS and case–control status remained when childhood adversity was added into the model (OR 1.79; 95% CI 1.53–2.10; *p* < 0.001).

### Gene–environment correlation

To test the possibility of gene–environment correlation, we examined the associations between SZ-PRS and childhood adversity, adjusting for PCs, site, sex, age and years of education. When each childhood adversity was analysed as a binary variable, no association was observed between SZ-PRS and childhood adversities in either cases or controls (*β*_case_ = 0.02; 95% CI −0.14 to 0.22; *p* = 0.65; *β*_control_ = 0.03; 95% CI −0.09 to 0.25; *p* = 0.34, respectively, see Table 3). Sensitivity analysis testing childhood adversities as a continuous score suggested a small but positive association with SZ-PRS in the controls, but not in cases (*β*_control_ = 0.09; 95% CI 0.02–0.16; *p* = 0.02; *β*_case_ = 0.02; 95% CI −0.08 to 0.11, *p* = 0.74, respectively, see online Supplementary Table S5). Dividing into subtypes of trauma, confirmed the above findings (see online Supplementary Table S6). Within controls only, a positive relationship was observed between SZ-PRS and emotional abuse (*β*_control_ = 0.09; 95% CI 0.02–0.15, *p* = 0.02).

**Table 3.** Associations between the SZ-PRS and reports of childhood adversity

Gene–environment correlation	Adjusted *β*^a^	95% CI	*p*	Adjusted *β*^b^	95% CI	*p*
Psychosis cases	0.02	−0.14 to 0.22	0.65	0.02	−0.14 to 0.22	0.65
Unaffected controls	0.03	−0.09 to 0.25	0.34	0.04	−0.08 to 0.26	0.31

*Notes*: Linear regression.

Childhood adversity was measured by the CTQ dichotomised into at least one type of trauma reaching moderate to severe levels (Bernstein et al., 1994).

a Adjusted for 10 PCs, and site.

b Further adjusted for sex, age at interview and years of education.

### Synergistic effects of childhood adversity and polygenic risk in FEP

**Fig. 1.**
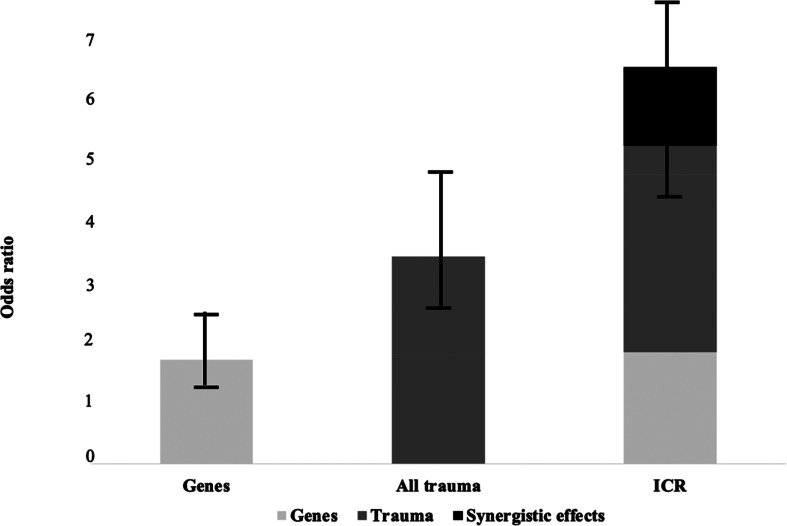
Additive effects of childhood adverse events and polygenic risk on case–control status.

The combined effect of childhood adversity (at least one type of trauma reaching moderate to severe levels) and polygenic risk was greater than the sum of each alone, but the CI included zero (ICR = 1.28, 95% CI −1.29 to 3.85; see Table 4, Fig. 1). Explorative analyses dividing into subtypes of childhood adversity showed the largest ICR for physical abuse (ICR = 6.25, 95% CI −6.25 to 20.88) and physical neglect (ICR = 3.68, 95% CI −1.69 to 9.06; see Table 5). The ICR was above zero for physical abuse, emotional abuse, emotional neglect and physical neglect, but CIs included zero for all analyses. Data were adjusted for site, sex, age and 10 PCs.

**Table 4.** Synergistic effects of childhood exposures and PRS-SZ_75_ on case status

	PRS-SZ_75_ = 0		PRS-SZ_75_ = 1		
	*N* cases/controls	OR (95% CI)	*N* cases/controls	OR (95% CI)	ICR (95% CI)
All trauma = 0	137/416	1.0	77/130	1.83 (1.26–2.65) *p* < 0.001	1.28 (−1.29 to 3.85)
All trauma = 1	100/104	3.38 (2.34–4.88) *p* < 0.001	71/43	5.33 (3.35–8.49) *p* < 0.001	

PRS-SZ_75_, polygenic risk score for schizophrenia (75% cut-point); ICR, interaction contrast ratio.

Data adjusted for site, sex, age and 10 PCs. Childhood adversity was measured by the CTQ dichotomised into at least one type of trauma reaching moderate to severe levels (Bernstein et al., 1994). ‘All trauma 0’ = no subtype of trauma reaching moderate to severe levels. ‘All trauma 1’ = at least one type of trauma reaching moderate to severe levels.

**Table 5.** Synergistic effects of childhood exposure subtypes and PRS-SZ_75_ on case status

	PRS-SZ_75_ = 0		PRS-SZ_75_ = 1		
	*N* cases/controls	OR (95% CI)	*N* cases/controls	OR (95% CI)	ICR (95% CI)
Emotional abuse = 0	194/487	1.0	122/161	1.92 (1.39–2.65) *p* < 0.001	0.58 (−4.29 to 5.46)
Emotional abuse = 1	43/33	4.34 (2.54–7.42) *p* < 0.001	26/12	5.89 (2.74–12.46) *p* < 0.001	
Physical abuse = 0	213/503	1.0	130/168	1.82 (1.33–2.48) *p* < 0.001	6.25 (−6.25 to 20.88)
Physical abuse = 1	24/17	4.34 (2.12–8.99) *p* < 0.001	218/5	12.08 (4.11–35.48) *p* < 0.001	
Sexual abuse = 0	204/492	1.0	139/1665	2.11 (1.55–2.89) *p* < 0.001	<0.1 (−6.20 to 0.15)
Sexual abuse = 1	33/28	3.91 (2.21–6.93) *p* < 0.001	9/8	2.00 (0.66–6.04) *p* < 0.001	
Emotional neglect = 0	184/467	1.0	108/149	1.81 (1.29–2.53) *p* < 0.001	1.21 (−1.79 to 4.20)
Emotional neglect = 1	53/53	2.85 (1.80–4.51) *p* < 0.001	40/24	4.86 (2.69–8.78) *p* < 0.001	
Physical neglect = 0	192/483	1.0	106/157	1.69 (1.21–2.35) *p* < 0.001	3.68 (−1.69 to 9.06)
Physical neglect = 1	45/37	3.71 (2.20–6.24) *p* < 0.001	42/16	8.08 (4.19–15.58) *p* < 0.001	

PRS-SZ_75_, polygenic risk score for schizophrenia (75% cut-point); ICR, interaction contrast ratio.

Data adjusted for site, sex, age and 10 PCs. Childhood adversity was measured by the CTQ dichotomised into at least one type of trauma reaching moderate to severe levels (Bernstein et al., 1994).

Follow-up analyses confirmed similar findings analysing SZ-PRS as a continuous variable. Since ICR was greater than zero, the combined effect of childhood adversities (at least one type of trauma reaching moderate to severe levels) and polygenic risk was larger than the sum of each alone (ICR = 1.24, 95%; CI −14.23 to 63.67).

## Discussion

We found independent effects of genetic liability and childhood adverse events in the onset of FEP, and some suggestive evidence that these factors combined synergistically to affect risk. The combined effect of childhood adversities (at least one type of trauma reaching moderate to severe levels) and genetic liability for schizophrenia was greater than the sum of each alone, but estimates lacked precision and CIs were wide, thus larger studies are needed before any firm conclusions can be drawn. Dividing into subtypes of childhood adverse events, explorative analyses revealed an ICR above zero for physical neglect, physical abuse, emotional abuse and emotional neglect. However, due to the very wide CI only tentative inferences can be drawn from these results. Synergistic effect of SZ-PRS and childhood adverse events has been recently reported in a larger sample of chronic schizophrenia, *N* = 1699 and 1542 unrelated controls (Guloksuz et al., 2019).

In line with previous findings, the SZ-PRS was associated with psychotic disorder in this large multi-centre study (Schizophrenia Working Group of the Psychiatric Genomics Consortium, 2014; Pardiñas et al., 2019). In addition, the cumulative effect of childhood adversity was associated with case/control status, consistent with previous studies of childhood adverse events and increased risk for psychosis (Church, Andreassen, Lorentzen, Melle, & Aas, 2017; Shevlin, Houston, Dorahy, & Adamson, 2008; Trotta et al., 2016), with higher OR in participants with multiple *v.* one type of trauma. Similar to the study by Trotta et al. (2016), we found no association between genetic liability for schizophrenia and childhood adversity assessed as a binary measure. However, sensitivity analyses suggested a small positive correlation between polygenic risk for schizophrenia and childhood adverse events in the unaffected controls, but not in the cases. Given that parental psychopathology may increase the likelihood of a child being maltreated (Sidebotham, Golding, & Parents, A. S. T. A. L. S. O., & Children, 2001), it could be that ‘the genetic substrate of the parents leads to both the abuse and to the illness in the children’ (Torrey, 2002), thus, in favour of a positive correlation. However, a complex interplay between a variety of factors is probably present, including, but not limited to factors that are not within the direct control of the individual (e.g. socioeconomic status). Our results are therefore partially consistent with these finding showing a correlation in the unaffected controls, but not in the cases. It could also be speculated that we had greater statistical power in the larger healthy control sample (*N* = 690), than the smaller patients' sample (*N* = 384) which could be reflected in the findings above. However, the high levels of childhood adversity in cases were not a consequence of genetic vulnerability in our sample.

As described by Trotta et al. (2016) childhood maltreatment may trigger maladaptive beliefs about the self and the world, including a negativity bias in attribution of others' intentions, disruption of the self and low personal control of events which may all trigger and maintain psychotic symptoms (Garety, Bebbington, Fowler, Freeman, & Kuipers, 2007; Howes & Murray, 2014; Rajkumar, 2014). As suggested in the stress-diatheses model (Pruessner, Cullen, Aas, & Walker, 2017; Walker & Diforio, 1997) exposure to childhood adversity may ‘sensitise’ an individual with a genetic risk for psychosis to later life stressors with exaggerated emotional responses and subsequent psychotic symptoms. This is supported by a recent study showing elevated hair cortisol (measure of stress over time) in psychotic adult patients with childhood adverse event experiences (Aas et al., 2019b), indicating long-term changes of the hypothalamic–pituitary–adrenal (HPA) axis following childhood adverse events. It has also been suggested that childhood adverse events and stress lead to an elevated dopamine function in the associative striatum (Deutch, Clark, & Roth, 1990; Egerton et al., 2016), which is relevant to positive symptom formation (Kapur, Mizrahi, & Li, 2005) and long-term changes in the HPA system following trauma events (Aas et al., 2019b). Our findings point to possible modest synergistic effects of genetic liability and childhood adversity experiences in the onset of FEP. However, these findings should be interpreted with caution as the CIs of the ICRs were large and included zero. It should also be noted that recent studies indicate independent risk of childhood adverse events and genetic risk in severe mental disorders (Aas et al., 2019a; Croft et al., 2019; Lecei et al., 2019), and due to the large variation of estimates within our study we cannot rule out the possibility of no effect.

### Limitations

Childhood trauma was reported retrospectively, with the inherent weakness of the retrospective design. A recent meta-analysis study suggests low overlap between retrospective and prospective collection of childhood trauma (Baldwin, Reuben, Newbury, & Danese, 2019). However, this study reported large heterogeneity within the meta-analysis. Albeit, it should be mentioned that reliance solely on retrospective assessment methods may have led to a proportion of non-exposed group being misclassified and thus affecting the results (Newbury et al., 2018; Reuben et al., 2016); therefore, these results should be interpreted with caution. As discussed by Knol and VanderWeele (2012), even though CIs include zero, if the additive estimate (here ICR) is above zero and CIs show a trend towards a positive interaction (skewed above zero), there is indicative evidence that the estimated effect on the additive scale is above zero and synergistic effects are present. A sample comprised of patients with an FEP consists of a wide range of individuals whose course of illness after the first episode can vary both in type of illness and in recovery. Using a more chronic homogenous group with a stable diagnosis may have yield greater ORs at least for the PRS. Furthermore, age at trauma exposure was not included as well as duration and frequency of the trauma which should be further investigated in future studies. The CTQ also does not cover all types of childhood trauma including questions on bullying; thus, some traumas may not have been picked up in this study.

To sum up, our study suggests that both a history of childhood adverse events and polygenic risk for schizophrenia modestly increase the risk for a psychotic illness, above that of childhood adverse events or polygenic risk alone. Thus, our findings indicate that experiencing childhood adverse events in individuals with high genetic risk for schizophrenia increases the likelihood of developing a psychotic illness more than individuals with low genetic risk for schizophrenia; however, the large CIs indicate that the findings should be interpreted with caution before replicated in larger independent samples.

## Supplementary material

For supplementary material accompanying this paper visit https://doi.org/10.1017/S0033291721003664. click here to view supplementary material
