# Supplementary material for: Synergistic effects of childhood adversity and polygenic risk in first-episode psychosis: the EU-GEI study
Source: Psychol Med. 2021 Sep 29;53(5):1970–8. doi: 10.1017/S0033291721003664 (PMC10106300; doi:10.1017/S0033291721003664)
Supplement: Supplementary file 1 [file S0033291721003664sup001.docx]

**Supplementary Material**

**Table S1.** Patients and controls divided into sites

| **site** | | | | | |
| --- | --- | --- | --- | --- | --- |
| Subject status | | | N | Percent % |  |
| Case |  | London | 38 | 9.6 |  |
|  |  | Cambridge | 34 | 8.6 |  |
|  |  | Amsterdam | 30 | 7.6 |  |
|  |  | Gouda and Voorhout | 67 | 16.9 |  |
|  |  | Madrid | 23 | 5.8 |  |
|  |  | Barcelona | 17 | 4.3 |  |
|  |  | Oviedo | 20 | 5.0 |  |
|  |  | Valencia | 21 | 5.3 |  |
|  |  | Creteil (Paris) | 12 | 3.0 |  |
|  |  | Puy de Dome (Clermont-Ferrand) | 6 | 1.5 |  |
|  |  | Bologne | 24 | 6.0 |  |
|  |  | Palermo | 13 | 3.3 |  |
|  |  | Sao Paulo WP2 | 22 | 5.5 |  |
|  |  | Galicia | 17 | 4.3 |  |
|  |  | Verona | 40 | 10.1 |  |
|  |  | Cuenca | 13 | 3.3 |  |
|  |  |  |  |  |  |
| Control |  | London | 86 | 12.3 |  |
|  |  | Cambridge | 90 | 12.8 |  |
|  |  | Amsterdam | 46 | 6.6 |  |
|  |  | Gouda and Voorhout | 74 | 10.5 |  |
|  |  | Madrid | 25 | 3.6 |  |
|  |  | Barcelona | 20 | 2.8 |  |
|  |  | Oviedo | 14 | 2.0 |  |
|  |  | Valencia | 14 | 2.0 |  |
|  |  | Creteil (Paris) | 39 | 5.6 |  |
|  |  | Puy de Dome (Clermont-Ferrand) | 32 | 4.6 |  |
|  |  | Bologne | 27 | 3.8 |  |
|  |  | Palermo | 29 | 4.1 |  |
|  |  | Sao Paulo WP2 | 62 | 8.8 |  |
|  |  | Galicia | 28 | 4.0 |  |
|  |  | Verona | 89 | 12.7 |  |
|  |  | Cuenca | 27 | 3.8 |  |
|  |  |  |  |  |  |

Patients and controls were recruited from 16 different sites as part of the EU-GEI study. (for more detailed information please see (Jongsma *et al.*, 2018).

**S2.** Flowchart.


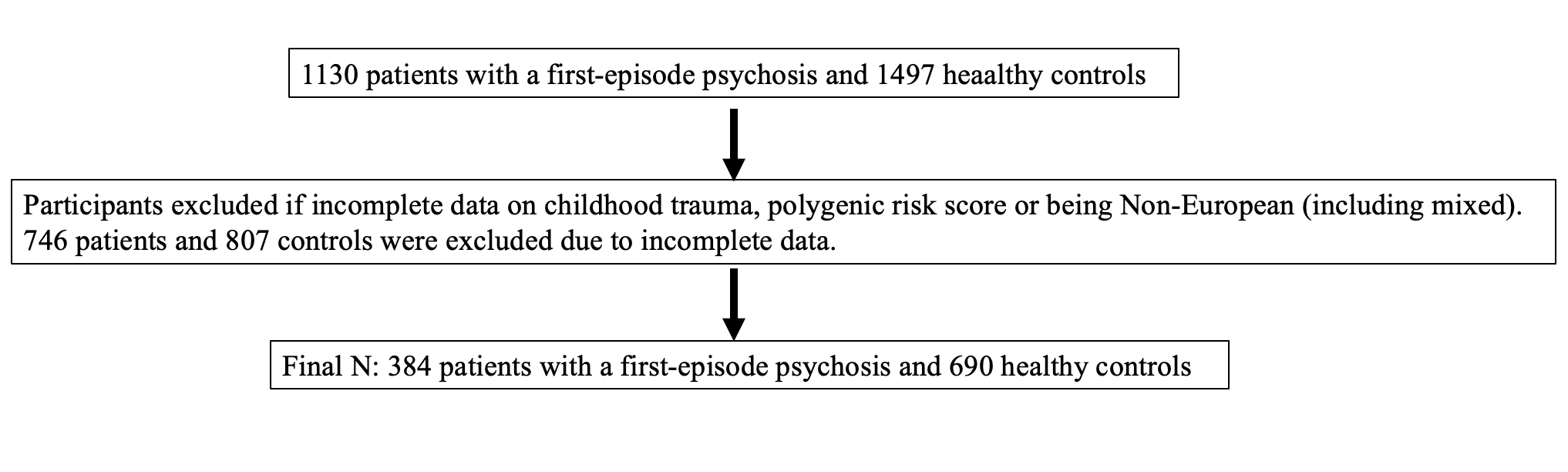


**Table S3.** Sensitivity analysis, prevalence of childhood adversities as a continuous measure amongst first-episode psychosis cases and unaffected controls

| **Total adversity exposure, CTQ**  **Total score** | **Unadjusted**  **ß** | \| **95% CI** \|  \| \| --- \| --- \| | ***p*** | **Adjusted ß ^*^** | **95% CI** | ***p*** |
| --- | --- | --- | --- | --- | --- | --- | --- | --- |
|  | 0.94 | 0.93-0.95 | ˂0.001 | 0.94 | 0.92-0.95 | ˂0.001 |

**Notes:** CI, confidence interval.

*Adjusted for site, gender, age at interview, and years of education.

Childhood adversities were measured as a continues score following the description by Bernstein *et al.,* (1994), with a minimum score of 25 and a maximum score of 125.

**Table S4.** Sensitivity analysis, prevalence of childhood adversities subtypes as a continuous measure amongst first-episode psychosis cases and unaffected controls

| **Emotional abuse** | **Unadjusted**  **ß** | \| **95% CI** \|  \| \| --- \| --- \| | ***p*** | **Adjusted ß ^*^** | **95% CI** | ***p*** |
| --- | --- | --- | --- | --- | --- | --- | --- | --- |
|  | 0.88 | 0.85-0.91 | ˂0.001 | 0.86 | 0.83-0.90 | ˂0.001 |
| **Physical abuse** | **Unadjusted**  **ß** | \| **95% CI** \|  \| \| --- \| --- \| | ***p*** | **Adjusted ß ^*^** | **95% CI** | ***p*** |
|  | 0.82 | 0.77-0.88 | ˂0.001 | 0.81 | 0.75-0.87 | ˂0.001 |
| **Sexual abuse** | **Unadjusted**  **ß** | \| **95% CI** \|  \| \| --- \| --- \| | ***p*** | **Adjusted ß ^*^** | **95% CI** | ***p*** |
|  | 0.87 | 0.81-0.93 | ˂0.001 | 0.86 | 0.79-0.93 | ˂0.001 |
| **Emotional neglect** | **Unadjusted**  **ß** | \| **95% CI** \|  \| \| --- \| --- \| | ***p*** | **Adjusted ß ^*^** | **95% CI** | ***p*** |
|  | 0.88 | 0.86-0.91 | ˂0.001 | 0.88 | 0.86-0.92 | ˂0.001 |
| **Physical neglect** | **Unadjusted**  **ß** | \| **95% CI** \|  \| \| --- \| --- \| | ***p*** | **Adjusted ß ^*^** | **95% CI** | ***p*** |
|  | 0.81 | 0.77-0.85 | ˂0.001 | 0.81 | 0.76-0.85 | ˂0.001 |

**Notes:** CI, confidence interval.

*Adjusted for site, gender, age at interview, and years of education.

Childhood trauma subtypes were measured as a continues score following the description by Bernstein *et al.,* (1994), with a minimum score of 5 and a maximum score of 25.

**Table S5.** Sensitivity analysis, schizophrenia polygenic risk score and reports of childhood adversity as a continuous measure

| **Gene–Environment correlation** | **Adjusted** **ß ^*^** | \| **95% CI** \|  \| \| --- \| --- \| | ***p*** | **Adjusted ß ^**^** | **95% CI** | ***p*** |  |
| --- | --- | --- | --- | --- | --- | --- | --- | --- | --- |
| Psychosis cases | 0.01 | -0.08-0.10 | 0.85 | 0.02 | -0.08-0.11 | 0.74 | |
| Unaffected controls | 0.08 | 0.03-0.15 | 0.02 | 0.09 | 0.02-0.16 | 0.02 | |

**Notes:** linear regression.

*adjusted for ten principal components, and site.

**further adjusted for gender, age at interview and education level.

Childhood adversities were measured as a continues score following the description by Bernstein *et al.,* (1994), with a minimum score of 25 and a maximum score of 125.

**Table S6.** Sensitivity analysis, schizophrenia polygenic risk score and reports of childhood trauma subtypes as a continuous measure

| **Gene–Emotional abuse correlation** | **Adjusted ß ^*^** | **95% CI** | ***p*** |  |
| --- | --- | --- | --- | --- |
| Psychosis cases | -0.004 | -0.09-0.10 | 0.94 | |
| Unaffected controls | 0.09 | 0.02-0.15 | 0.02 | |
| **Gene– Physical abuse correlation** | **Adjusted ß ^*^** | **95% CI** | ***p*** |  |
| Psychosis cases | 0.003 | -0.12-0.06 | 0.51 | |
| Unaffected controls | 0.06 | -0.01-0.13 | 0.10 | |
| **Gene–Sexual abuse correlation** | **Adjusted ß ^*^** | **95% CI** | ***p*** |  |
| Psychosis cases | -0.05 | -0.14-0.05 | 0.32 | |
| Unaffected controls | 0.07 | -0.05-0.13 | 0.07 | |
| **Gene– Emotional neglect correlation** | **Adjusted ß ^*^** | **95% CI** | ***p*** |  |
| Psychosis cases | 0.04 | -0.05-0.13 | 0.37 | |
| Unaffected controls | 0.06 | -0.01-0.13 | 0.11 | |
| **Gene–Physical neglect correlation** | **Adjusted ß ^*^** | **95% CI** | ***p*** |  |
| Psychosis cases | 0.04 | -0.06-0.13 | 0.44 | |
| Unaffected controls | 0.04 | -0.03-0.11 | 0.29 | |

**Notes:** linear regression.

* Adjusted for ten principal components, site, gender, age at interview and education level.

Childhood trauma subtypes were measured as a continues score following the description by Bernstein *et al.,* (1994), with a minimum score of 5 and a maximum score of 25.
